# A Biofeedback App for Migraine: Development and Usability Study

**DOI:** 10.2196/23229

**Published:** 2021-07-28

**Authors:** Sigrid Hegna Ingvaldsen, Erling Tronvik, Eiliv Brenner, Ingunn Winnberg, Alexander Olsen, Gøril Bruvik Gravdahl, Anker Stubberud

**Affiliations:** 1 Department of Neuromedicine and Movement Science Norwegian University of Science and Technology Trondheim Norway; 2 National Advisory Unit on Headaches Department of Neurology and Clinical Neurophysiology St. Olavs Hospital Trondheim Norway; 3 Department of Psychology Norwegian University of Science and Technology Trondheim Norway; 4 Department of Physical Medicine and Rehabilitation St. Olavs Hospital Trondheim Norway

**Keywords:** mHealth, headache, wearables, smartphone

## Abstract

**Background:**

Biofeedback is effective in treating migraines. It is believed to have a beneficial effect on autonomous nervous system activity and render individuals resilient to stressors that may trigger a migraine. However, widespread use of biofeedback is hampered by the need for a trained therapist and specialized equipment. Emerging digital health technology, including smartphones and wearables (mHealth), enables new ways of administering biofeedback. Currently, mHealth interventions for migraine appear feasible, but development processes and usability testing remain insufficient.

**Objective:**

The objective of this study was to evaluate and improve the feasibility and usability of an mHealth biofeedback treatment app for adults with migraine.

**Methods:**

In a prospective development and usability study, 18 adults with migraine completed a 4-week testing period of self-administered therapist-independent biofeedback treatment consisting of a smartphone app connected to wearable sensors (Cerebri, Nordic Brain Tech AS). The app included biofeedback training, instructions for self-delivery, and a headache diary. Two wearable sensors were used to measure surface electromyographic voltage at the trapezius muscle and peripheral skin temperature and heart rate at the right second fingertip. Participants were instructed to complete a daily headache diary entry and biofeedback session of 10 minutes duration. The testing period was preceded by a preusability expectation interview and succeeded by a postusability experience interview. In addition, an evaluation questionnaire was completed at weeks 2 and 4. Adherence was calculated as the proportion of 10-minute sessions completed within the first 28 days of treatment. Usability and feasibility were analyzed and summarized quantitatively and qualitatively.

**Results:**

A total of 391 biofeedback sessions were completed with a median of 25 (IQR 17-28) per participant. The mean adherence rate was 0.76 (SD 0.26). The evaluation questionnaire revealed that functionality and design had the highest scores, whereas engagement and biofeedback were lower. Qualitative preexpectation analysis revealed that participants expected to become better familiar with physical signals and gain more understanding of their migraine attacks and noted that the app should be simple and understandable. Postusability analysis indicated that participants had an overall positive user experience with some suggestions for improvement regarding the design of the wearables and app content. The intervention was safe and tolerable. One case of prespecified adverse events was recorded in which a patient developed a skin rash from the sticky surface electromyography electrodes.

**Conclusions:**

The app underwent a rigorous development process that indicated an overall positive user experience, good usability, and high adherence rate. This study highlights the value of usability testing in the development of mHealth apps.

## Introduction

Migraine is a very common disorder [1] and the leading cause for disability in people aged 15 to 55 years [2]. Frequent migraine attacks warrant preventive treatment, and nonpharmacological prophylaxes are a valid option, with few adverse events and a potential adjunctive effect to medication [3-5]. Several nonpharmacological interventions are proven to be effective, and among these, behavioral interventions are the most widely used [6]. Specifically, biofeedback is one of the most prominent behavioral approaches, and meta-analytical evidence suggests that it is effective in treating migraines [7].

During biofeedback, individuals learn to voluntarily modify their bodily reactions through feedback from their own physiological processes. The most frequently used modalities are peripheral skin temperature, blood volume pulse, and surface electromyography (SEMG) [8]. The exact mechanisms of the biofeedback training effect in migraine treatment are not known. It is believed to have a beneficial effect on autonomous nervous system activity, render individuals resilient to stressors, and possibly mediate a beneficial afferent vagus nerve stimulation [9]. Through regular training, individuals may learn a long-lasting reduction in muscle tension, rise in peripheral skin temperature, and lower heart rate. These measures are believed to be beneficial in reducing the migraine burden and are associated with increased parasympathetic tone [10,11]. Biofeedback treatment typically requires a trained therapist and specialized equipment measuring the chosen physiological parameter [8]. Today, biofeedback is primarily available in clinic-based formats, and to some extent in electronically delivered formats, and there is no clear evidence if the latter is inferior [12]. However, the need for a trained therapist and suitable equipment is costly and time-consuming, hampering the widespread use of biofeedback as a migraine prophylaxis.

Nevertheless, new digital technologies, including wearable sensors and smartphones for medical purposes (mHealth), provide new possibilities [13-15]. Recent research suggests that behavioral mHealth interventions for headaches are feasible, but development processes and usability testing remain insufficient [16]. Specifically, there is a lack of collaboration with health care professionals in the development process of pain apps [17-19]. Also, few existing mHealth pain apps use physiological and behavioral components that are often important factors for self-management interventions [20]. In addition to the limited development processes, efficacy measures are uncertain [12]. This study aimed to investigate the feasibility, usability, safety, and tolerability of a biofeedback treatment app and wearable sensors among adults with migraine.

## Methods

### Study Design and Participants

The study was designed as a prospective development and usability study at St. Olavs University Hospital in Trondheim, Norway, from December 2019 to March 2020. Adults with migraine were recruited from the outpatient headache clinic and the municipality using the hospital intranet and an advertisement in the news. A total of 18 participants were included in the study. We did not conduct a formal a priori sample size calculation. The sample size was based on recommended guidelines for a sample size of usability studies [21,22]. All diagnoses were confirmed by a consultant neurologist with headache expertise. Because there were only 10 pairs of sensors available for the study, participants were divided into two groups before completing a 4-week period of app testing at home (10 participants in the first group and 8 participants in the second group). The 4-week period was preceded by a preusability expectations interview and succeeded by a postusability experience interview. Participants also completed an evaluation questionnaire at weeks 2 and 4 of the test period. The study was approved by the regional committee for medical and health research ethics (REK Midt 7166) and the Norwegian Medicines Agency for trials of medical equipment (19/11730-9).

Inclusion criteria were aged 18 to 65 years; migraine with or without aura diagnosed according to the International Classification of Headache Disorders, Third Edition [23]; 2 to 8 attacks per month; experience with using a smartphone; and signed written informed consent. Exclusion criteria were lack of proficiency in the Norwegian language; reduced vision, hearing, or sensibility to a degree that hampered study participation; or any severe neurological or psychiatric disorders.

### Biofeedback Setup

**Figure 1:**
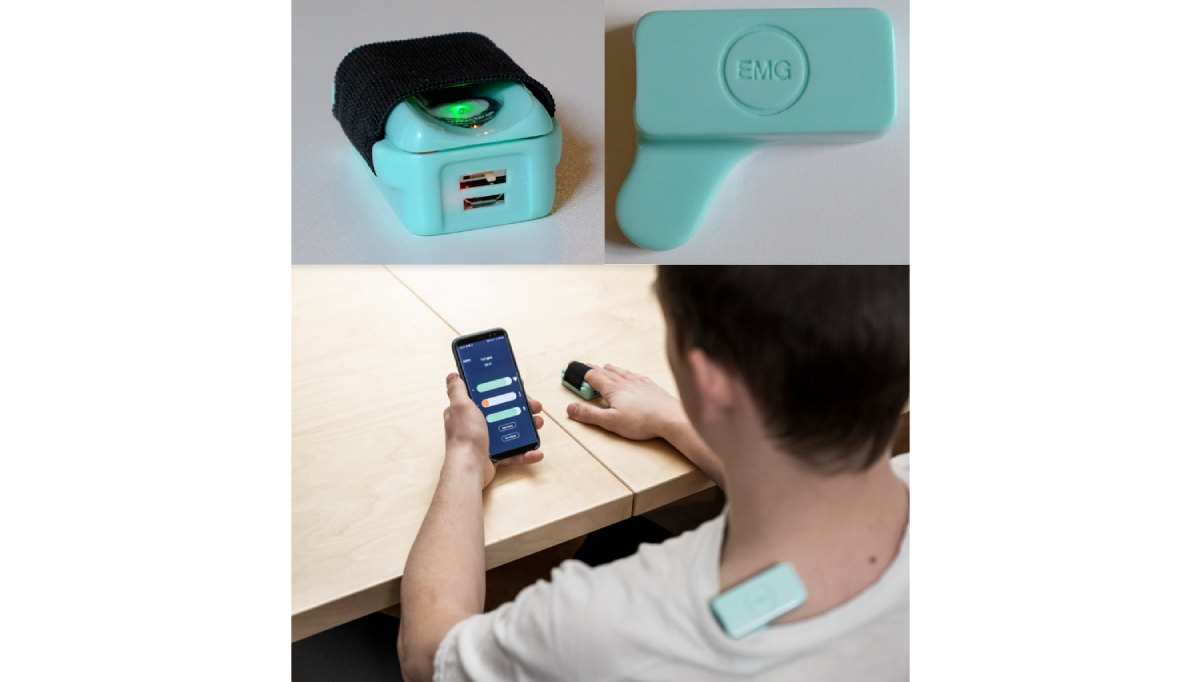
Photos of the wearable sensors. Top left: Combined sensor, measuring peripheral skin temperature and heart rate from the right index finger. Top right: Muscle tension sensor measuring surface electromyographic voltage from the upper trapezius muscle fibers. Bottom: Both sensors in use during a biofeedback session.

The Cerebri (Nordic Brain Tech AS) biofeedback setup consisting of a smartphone app connected to wearables was used for self-administered biofeedback treatment. The setup was developed based on similar equipment used in 2 previous studies at St. Olavs University Hospital. In the first of these studies, a biofeedback treatment app was developed in an iterative and incremental fashion, in which adolescents completed 3 cycles of usability testing, and improvements and changes to the app interface were completed between cycles [24,25]. A small compact SEMG sensor was used for measuring muscle tension from the upper trapezius muscle fibers. A combined device including 2 sensors was attached to the right index finger to measure peripheral skin temperature and heart rate (Figure 1 displays the sensors). Both devices transmitted signals to the app via Bluetooth Smart.

**Figure 2:**
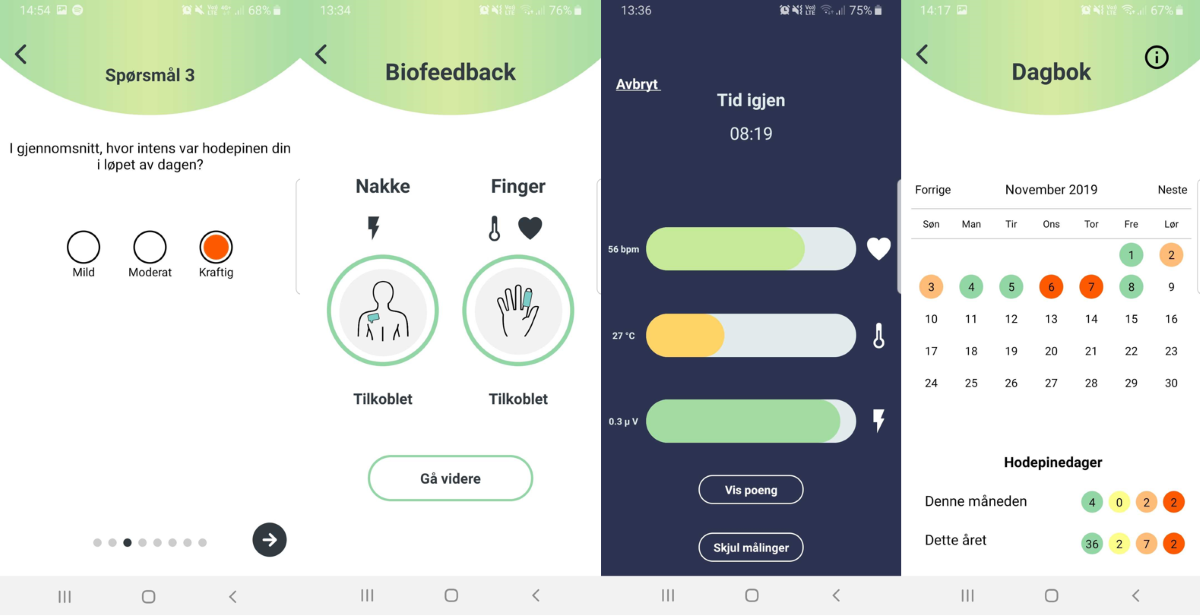
App screenshots. Left: Sample question in the headache diary where users are asked to rate headache intensity (On average, how intense was your headache during the day?). Center left: Instructions on how to connect the sensors. Center right: Visualization during the biofeedback session. Each of the three parameters is displayed as a horizontal column increasing in width with increasing score and correspondingly changing color. Right: Headache diary overview allowing for easy visualization of previous headache diary entries.

The app included biofeedback training, instructions for self-delivery, and a headache diary. Participants were given a push reminder daily to complete a headache diary entry and biofeedback session of 10 minutes. Prior to commencing treatment, participants were provided with a brief explanation of how biofeedback treatment works and how to complete the biofeedback training sessions. One of the investigators helped participants run through a trial session using both sensors and the app. Participants were encouraged to try to relax and sit comfortably during the sessions. No detailed instructions on how to perform the biofeedback were given. Figure 2 displays screenshots of the app. All participants were provided with a box containing both sensors, a power charger, and additional SEMG electrode patches. Participants used their own smartphones throughout the trial.

### Usability Evaluation

Upon inclusion, participants met with one of the researchers to download and start using the app. A semistructured preusability expectations interview was completed (Multimedia Appendix 1) before starting a 4-week period using the app and sensors.

During the 4 weeks, participants were prompted to complete daily biofeedback sessions and daily headache diary entries (Multimedia Appendix 2). Participants were given an evaluation questionnaire at 2 weeks and 4 weeks after commencing use (Multimedia Appendix 3). The evaluation questionnaire was similar to the one used in a recent adolescent usability study of a similar biofeedback setup [25]. The evaluation questionnaire included the following 5 main domains, engagement, functionality, design, information, and biofeedback, corresponding to the mobile app rating scale [26]. Questions were answered on a 5-point Likert scale, ranging from 1 = completely disagree to 5 = completely agree. Also, participants were encouraged to take notes of any adverse events and discomforts during the treatment period.

At the end of the 4-week home use period, participants met with one of the researchers to return the equipment and complete a semistructured postusability experience interview (Multimedia Appendix 1). At this point, participants were explicitly asked to report any skin reactions, dizziness, and nausea and openly questioned on other adverse events. All adverse events were recorded with their seriousness and the potential grade of causality.

### Data Analytic Strategy

We reported the number of completed sessions and calculated the adherence rate as the proportion of sessions completed within the first 28 days. Only sessions in which the full 10 minutes of biofeedback training was finished were considered as completed sessions. Sessions were automatically marked as completed within the app software, and the information was transferred to the database. No self-reported measure of adherence was made. We also described what weekdays and what time of the day sessions were completed.

Self-reported overall hours of phone use and familiarity with apps and sensors were averaged over the 2 evaluation questionnaires for each participant. Scores were averaged over each domain for all participants for the week 2 and week 4 evaluation questionnaires and summarized with medians and interquartile ranges. We only used complete data in the evaluation questionnaire analyses.

All usability interviews were recorded on an Olympus WS-853 recorder (Olympus America Inc). All recordings were transcribed and coded using NVivo 12 (QSR International) and stored in the software for qualitative analyses. A general inductive method was used to code transcripts. The transcripts were read repeatedly, and text segments were coded for potential themes. We used thematic content analysis to assess both preusability expectations and postusability experience [27]. We performed an inductive thematic content analysis for the expectations usability interview by generating codes that emerged naturally based on the participant responses to the semistructured interview guide. For the postuse usability interview, we conducted a problem-based deductive thematic content analysis to assess patterns of experience with the app and sensors and potential technical difficulties with the equipment. As the coding framework developed, transcripts were reanalyzed in light of new themes that emerged. Finally, we derived and summarized major themes that were relevant to the usability experience.

This is the primary analysis of data collected in this study. A priori, we planned for exploratory descriptive and qualitative analyses of usability data. No a priori hypothesis testing was planned, and none were conducted. Data were reported as means, standard deviations, medians, and interquartile ranges. Normality assumptions were based on visual inspection of histograms. Descriptive statistics were calculated, and figures were made using Python v3.7.7 (Python Software Foundation) with the following open-source packages: matplotlib 3.1.1, NumPy 1.17.2, pandas 0.20.3, and seaborn 0.9.0.

## Results

### Participants and Demographics

A total of 18 participants were recruited, attended, and completed the preusability and postusability evaluations. All participants had prior experience using health or wellness apps (such as headache diaries or meditation apps). Patient demographics are summarized in Table 1. Headache diary entry and biofeedback session data were not successfully transmitted to the data storage server for one participant and were thus not available for analyses.

**Table 1:** Patient baseline demographics.

Characteristic		Value (n=18)
Age (years), mean (SD)		40.6 (9.8)
Gender, female, n (%)		17 (95)
**Migraine subtype (n=17)**		
	Migraine with aura, n (%)	7 (41)
	Chronic migraine, n (%)	3 (18)
**Comorbid headache disorders**		
	Tension type headache, n (%)	7 (39)
	Trigeminal autonomic cephalalgias, n (%)	1 (6)
Self-reported monthly headache attacks, median (IQR)		4 (3.3-5)
Hours of daily smartphone use, mean (SD)		2.9 (1.0)
Familiarity with apps scored on a 5-point scale, median (IQR)		4 (4.0-4.25)
Familiarity with sensors scored on a 5-point scale, median (IQR)		1 (1-3)

### Usability Metrics

#### Use Patterns

A total of 391 biofeedback sessions for 17 individuals were completed, with a median of 25 (IQR 17-28) per participant. The mean adherence rate was 0.76 (SD 0.26). Session completion was evenly spread through the week with 50, 56, 54, 62, 57, 56, and 56 sessions completed on Monday, Tuesday, Wednesday, Thursday, Friday, Saturday, and Sunday, respectively. More than 90% (358/391, 91.56%) of sessions were completed between 4 PM and 11 PM, and 52.43% (205/391) were completed between 7 PM and 10 PM.

#### Preusability Expectations Interview

**Figure 3:**
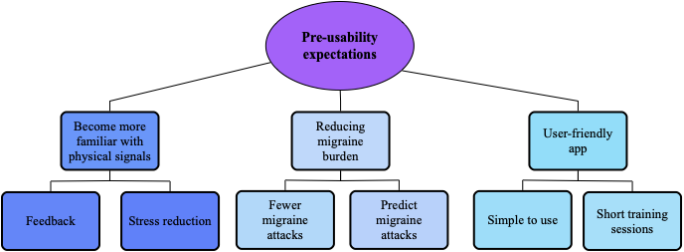
Inductive thematic content analysis of preusability expectations. Three major themes with several subthemes were found: becoming more familiar with physical signals, reducing migraine burden, and user-friendly app.

Coding of the preusability expectation interviews revealed 3 distinct major themes: becoming more familiar with physical signals, reducing migraine burden, and user-friendly app. Figure 3 is a diagram with an overview of themes and subthemes.

Almost all participants expressed a desire for the biofeedback sessions to help them become more familiar with bodily signals to understand what influenced their migraine and headache. Several participants had previously tried various relaxation and mindfulness exercises and reckoned the benefit of visualized feedback alongside such exercises. They expected that the explicit feedback in the app would enhance their motivation to use the equipment every day because it would show progress over time. Additionally, participants recognized the impact of stress on their migraine and thought the training would help them control and reduce stress levels.

All participants expressed that the main expectation of participating in the study was to reduce the migraine burden and improve their quality of life. Participants expressed that they wanted to understand their migraine better, learn to predict migraine attacks, and understand what triggered attacks. Some participants also noted that it would be interesting to see differences in the measurements on headache days compared to headache-free days.

Finally, as the third major theme, participants expressed the importance of a user-friendly app. The simplicity of use was essential if they were to use it while experiencing a headache so that the app would not worsen their headache. Some participants mentioned that short sessions would be beneficial due to the time constraints of a busy everyday life and the fact that the disease already consumes large amounts of their time.

#### Postusability Experience Interview

**Figure 4:**
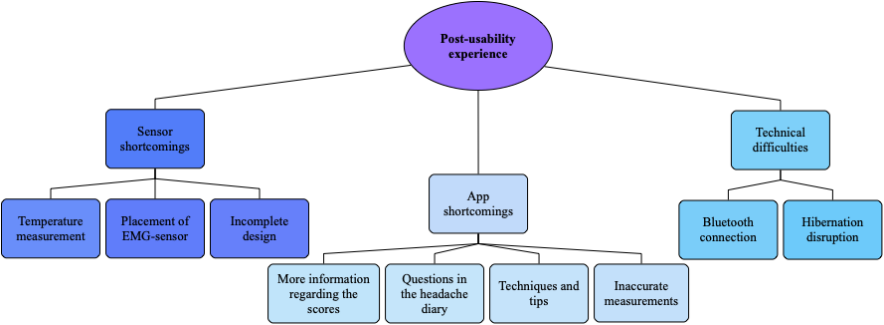
Deductive thematic content analysis of postusability experience. Three major themes with several subthemes were defined: sensor shortcomings, app shortcomings, and technical difficulties.

To assess postusability experience from a deductive problem-based approach, we used 3 major themes: sensor shortcomings, app shortcomings, and technical difficulties. The themes were based on the usability evaluation questionnaire (Multimedia Appendix 3) and interview answers. Inductive coding within each major theme revealed several subthemes (Figure 4).

Several of the participants felt that the temperature measurement did not reflect their skin temperature accurately. They also had difficulties understanding the association between finger temperature and stress/relaxation. Next, some participants had difficulties attaching the EMG sensor and found it too bulky to lie down and relax while performing the biofeedback sessions. Finally, some participants noted that the sensor design appeared prototype-like and that it might be easier to commercialize the sensors with a slimmer design.

Some participants wished for additional questions in the headache diary, such as associated symptoms and details on medication, to identify patterns in their migraine. Next, the app included limited information on biofeedback scores, and some participants found this information insufficient to understand the association between biofeedback performance and migraine burden. Some wanted an explicit overview of their scores and the direct association with headache occurrences, and one participant suggested an illustrative graph of score progression for each physiological measurement. Next, the app did not include specific instructions on biofeedback training, but several participants expressed a wish for relaxation techniques and/or tips to be included in the app. Finally, several participants said that the measures were inaccurate compared to how they felt. Some participants experienced that the measurements showed low scores on migraine-free days but high scores on days with migraine.

Some of the participants experienced technical difficulties. They had difficulties connecting the sensors to the app via Bluetooth and had to repeatedly switch sensors on and off during a session, which reduced the quality of the sessions. Additionally, several participants experienced that the session terminated early as their smartphone went into hibernation mode.

**Figure 5:**
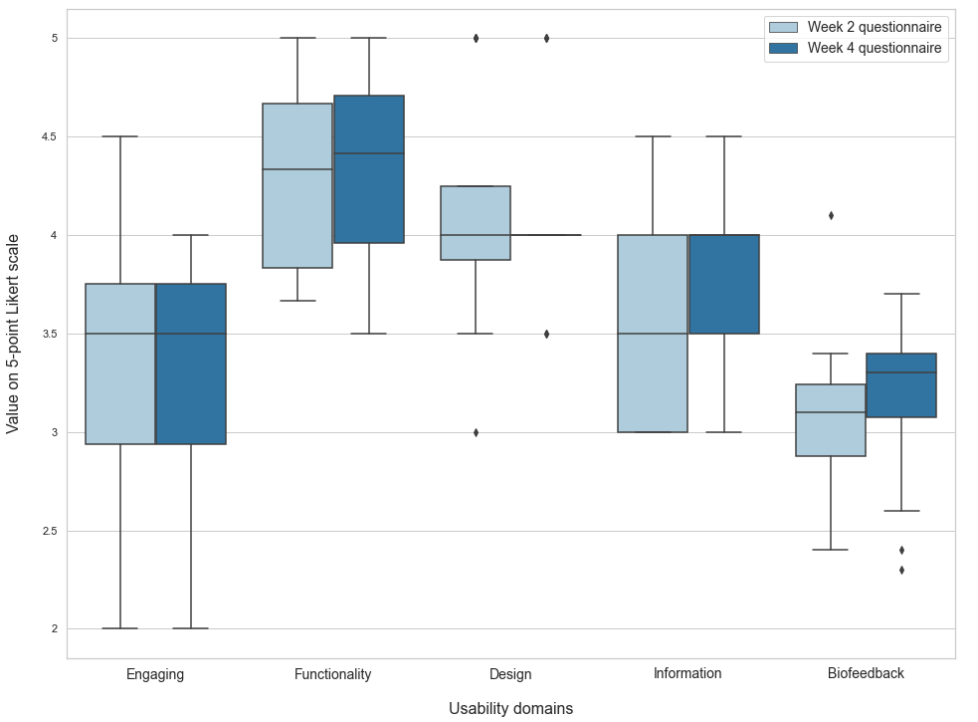
Boxplot of evaluation questionnaires. Horizontal lines represent medians, upper and lower box limits represent IQR, whiskers represent IQR*1.5, and diamonds represent outliers. Each pair of boxes shows the usability domain score after 2 (light blue) and 4 (dark blue) weeks of use. Note that functionality and design have the highest scores, whereas engagement and biofeedback scores are lower.

In addition to the thematic content analysis, results from the evaluation questionnaire illustrated postusability experience. The themes functionality and design showed the highest scores, whereas engagement and biofeedback scores were lower (Figure 5). A detailed visual presentation of the biofeedback ratings is provided in Multimedia Appendix 4.

### Safety and Tolerability

One case of prespecified adverse events was recorded, in which a patient developed a skin rash from the sticky SEMG electrodes. The rash was mildly painful and lasted for a week. No other adverse events were detected. Based on the evaluation questionnaire and qualitative analysis, some of the participants raised a discomfort concern regarding the bulkiness of the SEMG sensor when trying to lay down.

## Discussion

### Principal Findings

This study explored the usability and feasibility of a biofeedback app treatment for migraine using a mixed methods approach with both quantitative and qualitative data. The preusability assessment indicated that all participants were positively attuned to the biofeedback app and expected to learn more about their bodily signals and how these affect their migraine. On the other hand, the problem-based postusability analysis revealed that the setup had several shortcomings, including technical difficulties and inaccurate measurements. Most participants had some recommendations for specific improvements to the setup but reported an overall positive user experience and good adherence rates.

### Interpretation of Findings

Three main findings in this study illustrate the usefulness of usability testing when developing mHealth interventions. First, qualitative analysis revealed that participants felt they had difficulties with increasing their skin temperature. This is noteworthy because peripheral skin temperature appears to be one of the most effective biofeedback parameters for migraine [7,10] and is believed to provide an indirect measure of activity in the sympathetic nervous system, partially explaining the treatment effect of biofeedback [9,10,28]. A reduction in arousal and autonomic tone leads to increased peripheral blood flow and skin temperature, while increased arousal increases sympathetic outflow, thereby constricting peripheral blood flow and lowering skin temperature [29]. Migraine patients seem to have an interictal sympathetic impairment and ictal adrenoreceptor hypersensitivity [30], which suggest that regular training in reducing sympathetic tone could lower migraine burden [9]. Therefore, the importance of finger temperature in biofeedback training indicates that the perceived lack of influence over temperature lies in the app/sensor itself and not in the choice of the physiological parameter. An explanation for the mentioned difficulties with raising their finger temperature could be that the biofeedback sessions were completed during the cold winter in Norway. Thereby, the participants’ index finger might have been colder than the average body temperature prior to the biofeedback sessions.

Second, our mixed methods approach revealed that participants required more guidance to understand the association between biofeedback and the app’s illustration of the physiological measurements. The quantitative evaluation questionnaires revealed high scores for functionality and design but low scores for engagement and biofeedback, both in line with qualitative findings. Preexpectation analysis indicated that participants wished for the app to be simple and understandable. However, the postexperience analysis revealed that participants had difficulties perceiving how biofeedback is associated with migraine and better health outcomes. This suggests that upcoming iterations of the treatment should be focused on ensuring a tight correlation between users’ perception of the biofeedback training and true physiological measurements. This is likely to increase motivation and potentially treatment effect further [31].

The third significant usability finding was the desire for relaxation training and stress management techniques to be included in the app. We decided not to include such features to investigate if a therapist might be omitted from the usual treatment and see if the app itself may replace the therapist [25]. However, both qualitative and quantitative findings indicated that more guidance would have improved user experience and adherence. On the other hand, previous studies investigating the use of minimal therapist contact treatments have discovered that they often generate results equivalent to therapist-led treatment [32] and are more cost-effective [33]. Andersson and colleagues [34] investigated the role of therapist-initiated contact in a telephone study where adults suffering from headaches were randomized to either a web-based self-help program or the same program with additional therapist-initiated telephone calls. They found that therapist-initiated phone calls did not influence the dropout rate or improvements in headache index. This indicates that therapist-assisted treatment is not necessarily superior to pure self-management. The immediate idea to counteract this uncertainty in app-based biofeedback treatment is to implement a combination of home training and therapist contact. The problem, however, with such an approach is that it is not much different from traditional treatment and will not ensure the desired cost-benefit and widespread distribution. To keep in line with trends of mHealth development, we propose a program where minimal to no guidance is included in the app for the first few sessions, and then specific tips and techniques are progressively included for participants who have problems with achieving improvement in their biofeedback scores.

As the mHealth approach to pain treatment has grown over the past year, several shortcomings in development processes have been identified. Lalloo and colleagues [16] found that only 8.2% of pain self-management apps included a health care professional in the development and the majority of the apps (58.5%) implemented only a single self-management function. Similarly, Rosser and Eccleston [20] found that 86% reported no involvement of health care professionals in the development process. This could explain some of the general deficiencies they found, such as a lack of psychological and behavioral components underlying many self-management interventions, thereby causing an absence of validated expertise and content underpinning the available pain apps [20]. We took several measures to combat these limitations by including a wide range of health care professionals, including medical doctors and psychologists, in the development process.

Even though there are no similar studies of biofeedback for migraine, development studies of biofeedback for other purposes highlight the importance of a thorough development processes. A study of a sensor-based exercise biofeedback system found that using a systematic combined quantitative and qualitative assessment improved the system [35]. Another study of a mHealth biofeedback device for borderline personality disorder was also significantly improved through user-centered design with usability assessment [36]. Together with this study, both of these studies demonstrate that meticulous usability and feasibility assessments can mitigate the unwanted effects of poor development processes that often hamper mHealth apps [37].

### Limitations and Strengths

Several limitations should be considered when interpreting the results of this study. First, participants used the equipment over a relatively short period, whereas the International Headache Society recommends 3 months for clinical trials [38]. Second, each group of participants only completed one cycle of usability testing. The study could have benefitted from iterative cycles with several rounds of testing for each individual to identify the effect of improvements in the app directly [39]. This was not possible at the time because there were only 10 available sensors for the study. Finally, the semistructured interview guide and evaluation questionnaire used in the study have not been systematically validated, which decreases confidence in usability findings. Nevertheless, the interview guide was based on recommended guidelines for the development of mHealth apps [18], and the evaluation questionnaire was based on a validated mHealth app rating scale [40].

The study also has several strengths. The greatest strength is the rigorous usability and feasibility development process. As discussed above, we believe this will mitigate the unwanted effects of poor development processes. In addition, the study was designed based on recommended guidelines for the development of mHealth apps and known shortcomings in existing mHealth usability studies. Finally, although the sample size was seemingly small, the authors feel that the study reached a level of data saturation with the high adherence rate and collecting quantitative and qualitative data from all participants. We believe this study has uncovered the majority of essential usability issues.

### Conclusion

In this study, we performed usability and feasibility testing of a new biofeedback treatment app targeted at adults with migraine. The treatment underwent a rigorous development process specifically for the target population. Participants were overall satisfied with the treatment, had a high adherence rate, and provided several suggestions worthy of inclusion in future iterations. Our findings highlight the importance of usability testing, revealed shortcomings with the intervention that would otherwise have been difficult to discover, and built a solid foundation for future efficacy trials. Future research should assess the efficacy of the proposed biofeedback treatment app among adults with migraine.

## Appendix

Multimedia Appendix 1 Semistructured interview guide for preusability and postusability (translated to English).

Multimedia Appendix 2 Headache diary questions.

Multimedia Appendix 3 Usability evaluation questionnaire.

Multimedia Appendix 4 Boxplot of evaluation questionnaires in the biofeedback domain. Horizontal lines represent medians, upper and lower box limits represent IQR, whiskers represent IQR*1.5 and diamonds represent outliers. Each pair of boxes show the score the usability domains after 2 (light blue) and 4 (dark blue) weeks of use.

## Abbreviations

mHealth: mobile health
NTNU: Norwegian University of Science and Technology
SEMG: surface electromyography
