# Supplementary material for: A Biofeedback App for Migraine: Development and Usability Study
Source: JMIR Form Res. 2021 Jul 28;5(7):e23229. doi: 10.2196/23229 (PMC8367148; doi:10.2196/23229)
Supplement: Multimedia Appendix 1 [file formative_v5i7e23229_app1.docx]

**Multimedia appendix 1.** Semi structured interview guide for pre- and post-usability (translated to English).

**Pre-study usability-interview**

The following two elements are discussed during the usability-interview, conducted before one month of use:

1. Expectations
   - What do you generally expect from a migraine treatment?
   - What do you expect from this biofeedback treatment for migraine?

1. Previous treatment
   - Have you ever tested cognitive treatments or relaxation techniques to manage your migraine?
   - Have you ever experienced effect from using this kind of preventive techniques?
   - Have you ever used, or do you use a headache diary (for example Hodepinedagboka or Migraine Buddy)?

**Post-study usability-interview**

The following seven elements are discussed during the usability-interview, conducted after one month of use:

1. Engagement

- Was the app entertaining?
- Was the app interesting to use?
- Was the app interactive?
- Do you think the app is suitable for the migraine population?

1. Functionality

- Have you experienced any noticeable errors or delays in the app?
- How was it to learn how to use the app?
- Is the navigation between screens and app-components appropriate/logical?

1. Esthetics

- Is the app-content organized in a suitable manner?
- Is the app-graphics of a satisfactory quality?
- What do you think about the app´s appearance?
- Are the coulours calming?

1. Information

- Are the information and instructions detailed and explicit enough?
- Did you experience that the app had spesific and attainable goals?
- Is the quality and quantity of information acceptable?
- Do you wish for more instructions or information included in the app?
- Based on your experience, does the app give an impression that it comes from a legitimate source?

1. Subjective experience

- Are the sensors user-friendly?
- Did you experience any discomfort while using the sensors?
- What is your overall impression of the app?
- What did you feel (what was your experience) after completing a session?

6. Usability at home

- At what time during the day did you complete the biofeedback sessions?
- Did you experience any disruptions during the sessions?
- How much time are you willing to devote to the app every day?

7. Biofeedback as a product

- After using the biofeedback-treatment, would you pay for this kind of product?
- Where would you want to buy the product?
- Which adjustments has to be implemented for you to want to buy the product?
- How important is it that your physician recommends the product?
- Would you recommend the product to others?
