# Supplementary material for: A Biofeedback App for Migraine: Development and Usability Study
Source: JMIR Form Res. 2021 Jul 28;5(7):e23229. doi: 10.2196/23229 (PMC8367148; doi:10.2196/23229)
Supplement: Multimedia Appendix 2 [file formative_v5i7e23229_app2.docx]

**Multimedia appendix 2.** Headache diary questions

| **#** | **Question** |
| --- | --- |
| 1 | Did you have a headache today? (No/Yes; answering ‘No’ terminates diary entry) |
| 2 | How intense was your worst headache today? (4-point scale, where 0 = ’no pain’ and 3 = ‘severe pain’) |
| 3 | How intense was your headache on average during the day? (4-point scale, where 0=’no pain’ and 3 = ‘severe pain’) |
| 4 | How have you functioned in your daily activities today? (4-point scale, where 0 = ’No difficulties’, 1 = ’Minor difficulties’, 2 = ’Medium difficulties’, and 3 = ’Major difficulties’. |
| 5 | Did you take any acute medication because of headache today? (No/Yes). |
| 6 | Did you experience any of the following symptoms/signs within the last 24 hours? (selection of zero or more of the 14 most common premonitory symptoms from scroll down menu^37^) |
| 7 | How much sleep did you get last night? (hours and minutes) |
| 8 | How many hours did you work out during the previous 24 hours (hours and minutes) |
