# Supplementary material for: A Biofeedback App for Migraine: Development and Usability Study
Source: JMIR Form Res. 2021 Jul 28;5(7):e23229. doi: 10.2196/23229 (PMC8367148; doi:10.2196/23229)
Supplement: Multimedia Appendix 3 [file formative_v5i7e23229_app3.docx]

**Multimedia appendix 3**. Usability evaluation questionnaire

**Usability questionnaire for biofeedback app**

Circle the alternative that best answers the question.

**General**

How many hours do you use your smartphone daily?: _______

How much have you used apps on your smartphone?

1. Very little **2**. Little **3**. Some **4.** Much **5**. Very much

How much have you used wearable devices to measure functions in you body (for example pulse or blood sugar?

**1.** Very little **2**. Little **3**. Some **4.** Much **5**. Very much

**Engaging**

I enjoyed using the app together with the sensors.

**1.** Completely disagree **2.** Disagree **3.** Unsure **4.** Agree **5.** Completely agree

The contents of the app were interesting.

**1.** Completely disagree **2.** Disagree **3.** Unsure **4.** Agree **5.** Completely agree

I would like to use the app again.

**1.** Completely disagree **2.** Disagree **3.** Unsure **4.** Agree **5.** Completely agree

I would recommend the app to others.

**1.** Completely disagree **2.** Disagree **3.** Unsure **4.** Agree **5.** Completely agree

**Functionality**

It was easy to connect the sensors with the app.

**1.** Completely disagree **2.** Disagree **3.** Unsure **4.** Agree **5.** Completely agree

The sensors were easy to use.

**1.** Completely disagree **2.** Disagree **3.** Unsure **4.** Agree **5.** Completely agree

The app was easy to use.

**1.** Completely disagree **2.** Disagree **3.** Unsure **4.** Agree **5.** Completely agree

The app responded quickly.

**1.** Completely disagree **2.** Disagree **3.** Unsure **4.** Agree **5.** Completely agree

Navigating between screens and functions was easy.

**1.** Completely disagree **2.** Disagree **3.** Unsure **4.** Agree **5.** Completely agree

The app was unnecessarily complicated.

**1.** Completely disagree **2.** Disagree **3.** Unsure **4.** Agree **5.** Completely agree

**Design**

I liked the appearance of the app.

**1.** Completely disagree **2.** Disagree **3.** Unsure **4.** Agree **5.** Completely agree

The components included in the app (for example buttons, icons, content) is well organized?

**1.** Completely disagree **2.** Disagree **3.** Unsure **4.** Agree **5.** Completely agree

Regarding the appearance of the app, what did you like?

______________________________________________________________________________________________________________________________________________________________________________________________________________________________________________________________________________

__________________________________________________________________________________________

Regarding the appearance of the app, what did you not like?

______________________________________________________________________________________________________________________________________________________________________________________________________________________________________________________________________________

__________________________________________________________________________________________

**Information**

The app had enough information to be easy to use.

**1.** Completely disagree **2.** Disagree **3.** Unsure **4.** Agree **5.** Completely agree

I felt I could achieve the goals presented in the app (for example se changes in muscle tension and temperature and using the headache diary).

**1.** Completely disagree **2.** Disagree **3.** Unsure **4.** Agree **5.** Completely agree

**Biofeedback**

The instructions I received before using the app was useful.

**1.** Completely disagree **2.** Disagree **3.** Unsure **4.** Agree **5.** Completely agree

I felt that changes in feedback reflected my muscle tension.

**1.** Completely disagree **2.** Disagree **3.** Unsure **4.** Agree **5.** Completely agree

I felt that changes in feedback reflected my heart rate.

**1.** Completely disagree **2.** Disagree **3.** Unsure **4.** Agree **5.** Completely agree

I felt that changes in feedback reflected my finger temperature.

**1.** Completely disagree **2.** Disagree **3.** Unsure **4.** Agree **5.** Completely agree

The duration of the sessions was to long.

**1.** Completely disagree **2.** Disagree **3.** Unsure **4.** Agree **5.** Completely agree

I wish that the feedback from the three measures were combined.

**1.** Completely disagree **2.** Disagree **3.** Unsure **4.** Agree **5.** Completely agree

I liked how the feedback was presented on the screen.

**1.** Completely disagree **2.** Disagree **3.** Unsure **4.** Agree **5.** Completely agree

The biofeedback sessions helped me relax.

**1.** Completely disagree **2.** Disagree **3.** Unsure **4.** Agree **5.** Completely agree

I experienced improvements in my health over the course of the biofeedback sessions.

**1.** Completely disagree **2.** Disagree **3.** Unsure **4.** Agree **5.** Completely agree

How would you like the feedback to be presented on the screen (for example only one column, one curve, a picture that changes colours etc.):

______________________________________________________________________________________________________________________________________________________________________________________________________________________________________________________________________________

__________________________________________________________________________________________

**Safety**

I experienced discomfort using the app.

**1.** Completely disagree **2.** Disagree **3.** Unsure **4.** Agree **5.** Completely agree

I experienced discomfort using the sensors.

**1.** Completely disagree **2.** Disagree **3.** Unsure **4.** Agree **5.** Completely agree

If you experienced discomfort, please describe:

______________________________________________________________________________________________________________________________________________________________________________________________________________________________________________________________________________

__________________________________________________________________________________________

**General feedback and suggestions for adjustments**

What two things about the app did you enjoy the most?

______________________________________________________________________________________________________________________________________________________________________________________________________________________________________________________________________________

__________________________________________________________________________________________

______________________________________________________________________________________________________________________________________________________________________________________________________________________________________________________________________________

__________________________________________________________________________________________

What two things about the app did you not like?

______________________________________________________________________________________________________________________________________________________________________________________________________________________________________________________________________________

__________________________________________________________________________________________

______________________________________________________________________________________________________________________________________________________________________________________________________________________________________________________________________________

Describe with your own words your feelings when using the app, both physical and psychological:

________________________________________________________________________________________________________________________________________________________________________________________________________________________________________________________________________________________________________________________________________________________________________

______________________________________________________________________________________________________________________________________________________________________________________________________________________________________________________________________________

How can the app be improved to be more user friendly and fun to use?

________________________________________________________________________________________________________________________________________________________________________________________________________________________________________________________________________________________________________________________________________________________________________

______________________________________________________________________________________________________________________________________________________________________________________________________________________________________________________________________________

Do you think this app could be used for other purposes (if so, which ones)?

________________________________________________________________________________________________________________________________________________________________________________________________________________________________________________________________________________________________________________________________________________________________________

______________________________________________________________________________________________________________________________________________________________________________________________________________________________________________________________________________

Described with your own words what an app for migraine/headache should include:

______________________________________________________________________________________________________________________________________________________________________________________________________________________________________________________________________________

__________________________________________________________________________________________

______________________________________________________________________________________________________________________________________________________________________________________________________________________________________________________________________________

__________________________________________________________________________________________
