# Supplementary material for: A Biofeedback App for Migraine: Development and Usability Study
Source: JMIR Form Res. 2021 Jul 28;5(7):e23229. doi: 10.2196/23229 (PMC8367148; doi:10.2196/23229)
Supplement: Multimedia Appendix 4 [file formative_v5i7e23229_app4.docx]

**Multimedia Appendix 4.** Boxplot of evaluation questionnaires in the “Biofeedback” domain. Horizontal lines represent medians, upper and lower box limits represent IQR, whiskers represent IQR*1.5 and dimonds represent outliers. Each pair of boxes show the score the usability domains after two (light blue) and four (dark blue) weeks of use.

**
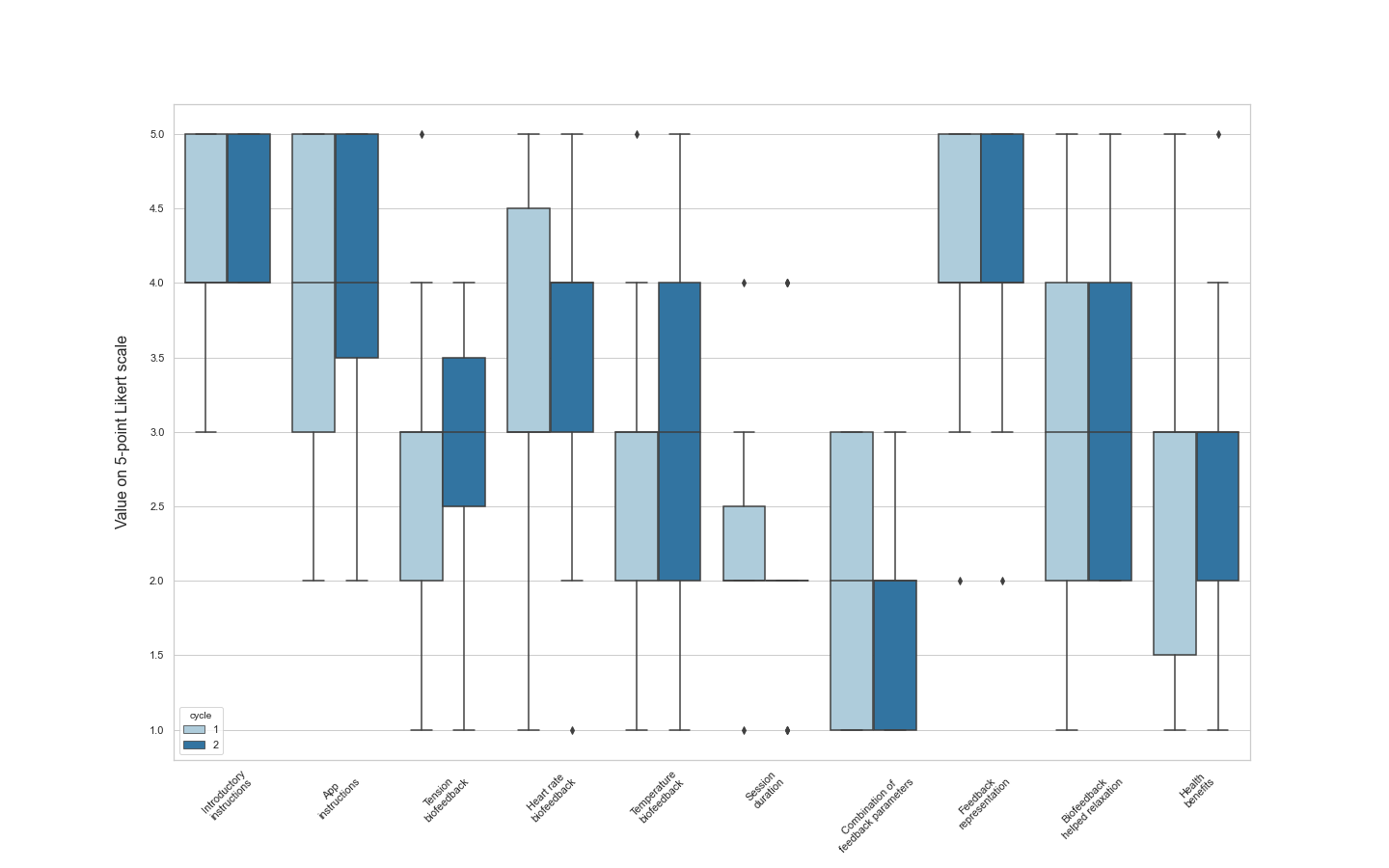
**
